# The off-line effect of affective touch on multisensory integration and tactile perceptual accuracy during the somatic signal detection task

**DOI:** 10.1371/journal.pone.0261060

**Published:** 2021-12-31

**Authors:** Sofia Sacchetti, Francis McGlone, Valentina Cazzato, Laura Mirams

**Affiliations:** School of Psychology, Liverpool John Moores University, Liverpool, United Kingdom; University G d’Annunzio, ITALY

## Abstract

Affective touch refers to the emotional and motivational facets of tactile sensation and has been linked to the activation of a specialised system of mechanosensory afferents (the CT system), that respond optimally to slow caress-like touch. Affective touch has been shown to play an important role in the building of the bodily self: the multisensory integrated global awareness of one’s own body. Here we investigated the effects of affective touch on subsequent tactile awareness and multisensory integration using the Somatic Signal Detection Task (SSDT). During the SSDT, participants were required to detect near-threshold tactile stimulation on their cheek, in the presence/absence of a concomitant light. Participants repeated the SSDT twice, before and after receiving a touch manipulation. Participants were divided into two groups: one received affective touch (CT optimal; n = 32), and the second received non-affective touch (non-CT optimal; n = 34). Levels of arousal (skin conductance levels, SCLs) and mood changes after the touch manipulation were also measured. Affective touch led to an increase in tactile accuracy, as indicated by less false reports of touch and a trend towards higher tactile sensitivity during the subsequent SSDT. Conversely, non-affective touch was found to induce a partial decrease in the correct detection of touch possibly due to a desensitization of skin mechanoreceptors. Both affective and non-affective touch induced a more positive mood and higher SCLs in participants. The increase in SCLs was greater after affective touch. We conclude that receiving affective touch enhances the sense of bodily self therefore increasing perceptual accuracy and awareness. Higher SCLs are suggested to be a possible mediator linking affective touch to a greater tactile accuracy. Clinical implications are discussed.

## 1 Introduction

Affective touch, or tactile processing with a hedonic or emotional component [1], is understood to be coded by specialised, unmyelinated mechanosensitive slow-conducting, peripheral nerves, known as C-tactile afferents (CT-afferents; [2, 3]). CT-afferents are mainly found in the hairy skin of the body [4, 5, but see [6] for recent evidence of CT in glabrous skin] and respond optimally to gentle stroking touch (1–10 cm/s stroking velocity; [7]. Specifically, responses of CT-afferents during single unit recordings with microneurography in response to different stroking velocities show that their spike discharge follows an inverted U-shaped pattern, with the greatest response at ~3 cm/s, and weaker responses at slower (0.1 cm/s) and faster velocities (30 cm/s; [7, 8]). Importantly, activation of CT-afferents correlates with the subjective rating of pleasantness, indicating that CT-afferents may constitute the peripheral physiological substrate for pleasant tactile information [7, 9, 10].

Because of their role in contributing to the hedonic value of social physical interactions, CT-afferents have been proposed to play a pivotal part in fostering affiliative behaviours and proximity seeking. Accordingly, different streams of research converging in the so called ‘Social Touch Hypothesis’ have recently underlined the importance of CT-afferents in promoting social bonding and attachment [1, 11]. Supporting this hypothesis, it has been shown that CT-afferents respond optimally to touch delivered at skin temperature [8]. Moreover, when asked to stroke babies or their partners, people spontaneously deliver touch at CT optimal velocities [12].

CT-afferents ascend via spinothalamic pathways to the posterior insula, a limbic area that is understood to support an early convergence of affective and sensory signals from the body [2, 13, 14]. In turn, remapping of information from the posterior to the anterior insula is thought to allow the integration of sensory signals with other bodily information as well as with other cognitive and social factors, ultimately serving body awareness and body self-consciousness [15, 16]. Indeed, this ascending neural path is hypothesised to be involved in the construction and maintenance of the so called ‘bodily self’, that is defined as a global, multimodal awareness of one’s own body, arising from the integration of information coming from different sensory modalities [17, 18].

Following this line of reasoning, researchers have investigated the role of affective touch in tasks assessing the integration of competing multisensory information such as the Rubber Hand Illusion (RHI; [19–22]). During the RHI, an experimenter strokes a visible rubber hand, that is placed in front of the participant, simultaneously to the participant’s own hand, that conversely is hidden from sight. The integration of contrasting visuo-tactile information induces many participants to perceive the rubber hand as their own (embodiment) and to mis-locate the position of their own hand as closer to the rubber hand (proprioceptive drift). Of relevance here is the finding that the illusion is strongest when the stroking touch is applied at CT-afferent preferred velocities (1–10 cm/s) vs non-affective touch stimuli, in terms of a higher embodiment (the misperception of the rubber hand as participants’ own hand [20, 22]) or in terms of a stronger proprioceptive drift (the mis-location of participants’ hand as closer to the rubber hand; [21]). The authors concluded that affective touch may have a unique contribution to the sense of body ownership as assessed using the RHI.

Similar results were found by Panagiotopoulou and colleagues [23] who investigated the effects of administering touch at CT optimal vs non-CT optimal velocities during the ‘enfacement illusion paradigm’. During this paradigm, participants are stroked on the cheek (a body site densely innervated by CT-afferents) whilst watching a video of another person’s face being stroked on the specular cheek. Subsequently, they are shown a video of the other person’s face gradually morphing with a photograph of their own face, and they are asked to say at what point the face looks more like them, than the other person. In agreement with previous research using the RHI, affective touch enhanced subjective self-face recognition during this task as indicated by participants’ self-reported experience of the illusion.

Taken together results of these studies suggest that in a multisensory body-awareness context, affective touch is perceived as more meaningful compared to a non-affective touch [3], leading participants to anchor their sense of bodily self to a greater extent to the affective tactile information mediated by CT-afferents, rather than other sensory information. This means that affective touch is potentially more relevant than discriminative touch information in building a global, multimodal perceptive model of one’s own body (bodily self). During multisensory integration tasks, such as the RHI and the enfacement illusion, this translates with affective touch enhancing the experience of body ownership [20, 23, 24].

Further support for the role of CT-afferents in generating the sense of bodily self comes from a positron emission tomography study (PET) which found that when contrasting affective touch on the forearm (high-CT innervated hairy skin) with affective touch on the palm (low-CT innervated glabrous skin) not only was there the previously reported activations in dorsal posterior insular cortex and mid-anterior orbitofrontal cortex, but there was also activation in the angular gyrus in the parietal cortex [3]. The angular gyrus has been shown by Blanke and colleagues [25] to trigger repeated out-of-body experiences when electrically stimulated in a study with an epilepsy patient. The activation in this area to CT-directed touch during the PET study poses an intriguing question as to the role of this area in coding for the sense of a bodily self.

However, in the studies mentioned above analysing the effects of affective touch on multisensory integration (i.e., the synthesis of sensory information from two or more sensory modalities to the building of a unitary body perception [26]), affective touch was an on-line active component of the tasks that participants were asked to perform, in the sense that affective touch was one of the concomitant and competing sensory information that participants were asked to elaborate and integrate in order to perform the task. Therefore, it was not possible to disentangle whether affective touch induced subsequent off-line alterations in multisensory integration. In a similar direction, a previous study by von Mohr and colleagues [27] indicated that affective touch can induce an emotional off-line effect in the form of a subsequent reduction in feelings of social exclusion. However, no studies to date have analysed whether a similar effect of affective touch can impact also subsequent multisensory integration. Information on this topic would be crucial in determining the potential for affective touch to be used as an intervention in clinical populations presenting with aberrant body perception and multisensory integration (i.e., people with eating disorders, body dysmorphic disorders, and medically unexplained symptoms).

In the current study, we investigated the effects of CT optimal (affective) vs CT non-optimal (non-affective) touch on subsequent multisensory integration using the Somatic Signal Detection Task (SSDT). The SSDT is a paradigm for the assessment of tactile perception and visuo-tactile multisensory integration. The SSDT involves detecting near-threshold vibrotactile pulses that occur on 50% of experimental trials, with and without a simultaneous spatially aligned flashing LED [28–30]. During this task, the presence of the light (LED) increases reports of feeling the touch when the tactile pulse is administered (as indicated by a significant increase in hit rates between light and no light trials). Alongside, the presence of the light also induces participants to erroneously report perceiving the touch when the tactile pulse was not administered (as indicated by an increase in false alarms between light and no light trials [28]). This effect of the light has been found to be a robust phenomenon consistent over time and it is therefore thought not to be influenced by learning mechanisms (e.g., learned association between the light and touch [31]).

In comparison to the RHI, and other paradigms assessing multisensory integration, the SSDT takes advantage of the use of Signal Detection Theory, which provides a more comprehensive description of participants responses, with separate measures of sensitivity (*d′*, i.e., the ability to correctly discriminate whether the tactile pulse was absent or present) and response criterion (i.e., the propensity to report feeling the tactile pulse regardless of the type of trial; [32]). Furthermore, it has been argued that participants’ responses during the RHI can be driven by suggestibility (demand characteristics), since during the illusion participants are aware of a distortion in their experience of an existing touch [33, 34]. Conversely, during the SSDT, participants are unaware of whether or not their experience of touch is accurate or distorted, and so this paradigm may be less susceptible to suggestibility and therefore be a more objective measure of tactile distortions due to multisensory integration. Moreover, it should be noted that the RHI aims to assess body ownership through the manipulation of multisensory information. Therefore, the RHI does not measure multisensory integration per se but rather it constitutes an indirect/implicit measure of multisensory processes, whereas the SSDT is a direct/explicit measure of multisensory integration.

As opposed to previous research in which CT optimal vs non-CT optimal touch was administered online as part of a multisensory task [20, 23], in the current study, the SSDT was performed offline: before and after receiving the touch. Indeed, previous research using the SSDT paradigm [35] has shown that offline interventions can have a carry-over effect to subsequent performance on the task. Specifically, focusing on interoceptive sensations (i.e., inner body signals) during a heart-beat perception task was found to increase participants’ reports of touch in the presence and absence of the target tactile pulse; conversely, focusing on external touch during a grating orientation task was found to decrease participants reports of touch [35]. Building on these results, in the current study, we investigated whether affective touch can also induce an offline carry-over effect on subsequent perceptual processes during multisensory integration measured by the SSDT.

Participants completed the SSDT twice before and after receiving either CT optimal vs non-CT optimal touch. Throughout the experiment, Skin Conductance Levels (SCLs) were also recorded to investigate whether the touch manipulation would induce changes in autonomic physiological arousal, and whether these changes would relate to SSDT responses. Furthermore, before and after receiving the touch manipulation participants were administered with self-report measures of mood (with a focus on Anxiety, Calmness, Happiness, Sadness).

Due to the functional role of CT-afferents in multisensory perception and body awareness, the main hypothesis of this study was that CT optimal touch would lead to a more accurate perception during the subsequent SSDT. More precisely, we predicted that affective touch would enhance perceptual awareness on the body site stimulated to a greater extent than non-affective touch. In turn, we expected this enhancement in perceptual awareness to enhance touch perception in that body site, leading participants to be better able to discriminate when the tactile pulse was administered and when it was not (higher sensitivity, *d’*). According to previous research that showed a unique link between affective touch and body awareness, we expected this effect to be specific for CT optimal touch as opposed to non-CT optimal touch [23, 24].

However, differently from previous research that assessed the effects of affective touch during the RHI, we expect CT optimal touch to enhance perceptual, tactile accuracy rather than amplify perceptual distortions. Indeed, during the RHI tactile information contributes to eliciting the perceptual illusion. Therefore, an enhancement in the salience of the tactile information (affective touch; i.e., CT optimal touch) determined a stronger perceptual distortion [20–22]. Conversely, during the SSDT, the presence of the touch (vibrotactile pulse) does not play a role in eliciting the illusion but rather it represents the genuine information required to detect in the absence of the perceptual illusion. Therefore, due to paradigm differences, in the current study we predicted affective touch to enhance perceptual accuracy rather than increase perceptual distortions. Moreover, as affective touch should enhance multisensory integration processes, we expected this manipulation also to increase the effect of the light on touch reports during the SSDT. Specifically, we expected participants receiving affective touch to be more accurate in detecting the tactile pulse during the SSDT (*d’*) especially in trials during which the light was present. Affective touch can be considered an interoceptive modality. However, hypotheses of this study are not built upon the fact that affective touch is mainly interoceptive vs exteroceptive but rather upon the fact that CT-afferents converge in a neural network responsible for body awareness and multisensory integration processes. For the purposes of this study, therefore, the nature of affective touch as an interoceptive vs exteroceptive modality has not been discussed.

Moreover, in line with previous studies that have investigated the effects of CT optimal touch using implicit measures of emotional valence (e.g., facial electromyography and physiological responses of skin conductance and heart rate variability; [36–38], we also hypothesized that CT optimal touch would increase positive mood (happiness and calmness) and decrease negative mood (anxiety and sadness).

## 2. Materials and methods

### 2.1 Design

Participants were tested in a single study session. All participants performed the SSDT at the beginning of the testing session, they then received a touch manipulation, and subsequently performed the SSDT a second time. Participants were randomly assigned to one of two possible touch manipulations: one group received CT optimal touch (3cm/s; n = 32), and the second group received non-CT optimal touch (30cm/s; n = 34). Therefore, the study employed a 2×2×2 mixed designed with group (CT optimal touch vs non-CT optimal touch) as a between subject variable, Light (Light vs No Light) and Time (Pre vs Post-touch) during the SSDT as within-subject variables, and hit rate (HR), false alarm rate (FA), *d*’ and *c* as dependent variables.

### 2.2 Participants

Sixty-six women between 19 and 60 years of age (*M* age = 32.67, *SD* = 15.42) were recruited from the staff and student population at Liverpool John Moores University (LJMU) and from the general population via advertisements placed around the university campus and on social media. The sample size was based on a power analysis using G*Power 3.1.9.7 [39], which indicated that overall a minimum sample of n = 56 was needed to detect a medium effect (*f* = .20) with 95% power, using a mixed design ANOVA (“number of groups” = 2 × “number of measurements” = 4) with alpha at .05 (two tailed). The sample size was expanded to 66 participants to increase statistical power (CT optimal group: n = 32; non-CT optimal group: n = 34).

Any individual who self-reported to have been previously clinically diagnosed with, or treated for body dysmorphic disorder (BDD) or eating disorders (EDs) were excluded from the study, as well as individuals who self-reported a history of, or any current neurological and/or psychiatric disorder. Further exclusion criteria included uncorrectable visual impairments, tactile impairments, skin conditions and pregnancy. To avoid any effect of gender differences on affective touch responses [40, 41], and consistent with previous studies by our research group [29, 30], we recruited only participants who self-identified as women. All participants but two were right-handed as assessed using the Edinburgh Handedness Inventory (EHI; [42]).

According to the Helsinki declaration of ethical standards, the study was approved by the LJMU’s Research Ethics Committee. All participants gave their informed consent to take part, and they were compensated for their time with a £5 shopping voucher or “participation points” for course credit for BSc Psychology students.

### 2.3 Material and measures

#### 2.3.1 The Somatic Signal Detection Task (SSDT)–face version [28, 29]

Participants sat in a light attenuated room approximately 40cm in front of a computer monitor (5:4 ratio; 270mm × 330mm). A tactor delivering vibrations (Z-Voom phones type YVE-01B-03, Yeil Electronics, South Korea; 1.8cm diameter) was fixed to participants’ left cheeks using double sided adhesive tape and a bandage tape to prevent movements. Tactile stimuli (20ms, 100Hz vibrations) were produced by sending amplified sound files (.wav files, sine wave), controlled by E-Prime software (Psychology Software Tools Inc., Pittsburgh, PA, USA), to the tactor.

**Fig 1:**
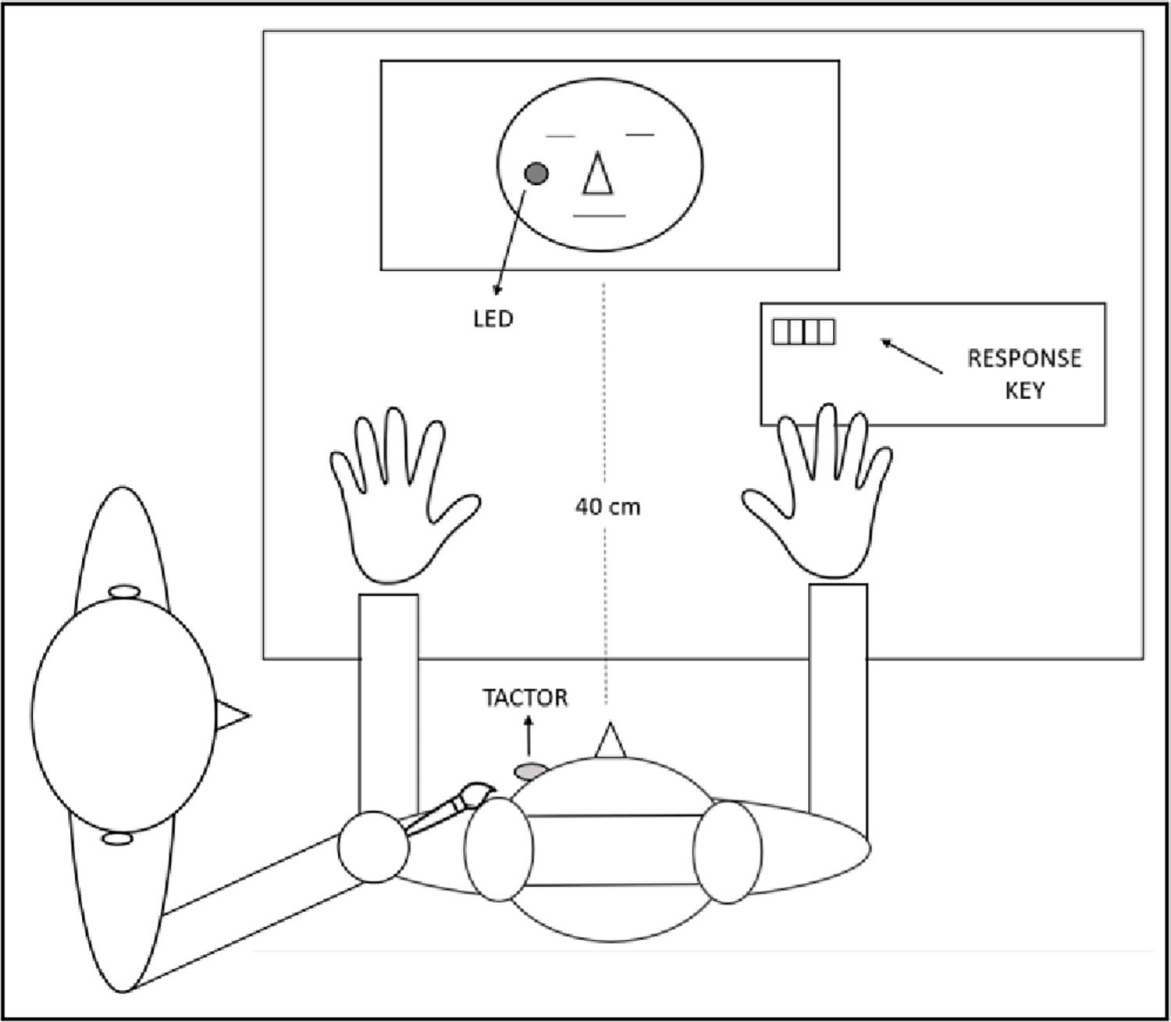
Schematic depiction of the experimental set-up during the SSDT. The experimenter (left) delivered the touch manipulation to the participant (right) between the first (Pre) and the second (Post) repetition of the SSDT.

A mirror-reversed photograph of the participant’s face (768 × 583 pixels in size) was presented on the computer screen during all trials of the SSDT. Instructions for participants were presented on the top section of the computer screen in order not to hinder vision of the photograph. During the experimental phase of the SSDT, a 4mm red light emitting diode (LED) was fixed to the computer monitor mirroring the location of the tactor on the participant’s face (see Fig 1). The LED was therefore placed on the right cheek of the face depicted on the monitor.

Gaze direction, and distance from the computer monitor during the task was controlled using a chin-rest that discouraged participants from moving their head. Throughout the experiment, participants listened to white noise via headphones to mask any informative sounds from the tactor. Underneath the headphones, participants wore a second pair of small earphones which administered auditory cues for signalling the beginning of each trial of the SSDT.

#### 2.3.2 Touch manipulation

During the touch manipulation, tactile stimulation (brush strokes) were delivered from a female experimenter using a cosmetic brush (No7 cosmetic brush, Boots UK). Prior to testing, the experimenter trained on a high precision scale to deliver strokes at a constant pressure of 220 mN (22gr/cm^2^; [43–45]). During the touch manipulation, participants were instructed to look at a blank screen presented on the computer monitor in front of them. As during the SSDT, movements of the head were prevented by the use of a chin-rest. The CT optimal group received strokes at the velocity of 3cm/s, and the non-CT optimal group at the velocity of 30cm/s. A visual metronome was presented on a computer screen behind the participant to guide the experimenter in delivering the strokes at the correct velocity (3cm/s or 30cm/s; [38, 46]). The metronome used a custom made PsychoPy script [47], showing a 3s countdown followed by a 9cm rectangle filling at the required stroking velocity (3s for the 3cm/s touch, and 0.3s for the 30cm/s touch).

The touch manipulation consisted of 4 blocks of 4 trials each, with each trial corresponding to a 6s window during which participants were stroked back and forth on a 9cm segment on their left cheek. Therefore, for the CT-optimal group (3cm/s) each trial consisted of 2 consecutive strokes administered, while for the non-CT optimal (30cm/s) each trial consisted of 20 strokes.

After each of the 4 blocks, participants were asked to rate the Pleasantness and Intensity of the stimulation on a 15cm Visual Analogue Scale (VAS) ranging from -10 (unpleasant) to +10 (pleasant), and from 0 (least intense) to 100 (most intense). Pleasantness and Intensity VASs were used as a manipulation check to ensure that CT optimal touch was perceived as more pleasant and less intense compared to non-CT optimal touch in accordance with previous research [e.g., 7, 8, 48].

Moreover, prior to, and after receiving the touch manipulation, participants were asked to report on four 15cm VASs how calm, anxious, happy, and sad they felt from a minimum of 0 (not at all) to a maximum of 100 (very much so). Mood VASs (Calmness, Anxiety, Happiness, Sadness) to analyse the influence of the touch manipulation on participants’ emotional state. VASs for psychosocial measurements have been shown to be a valid and reliable instrument [49]. In the current study, VASs were preferred to other available measures of mood because they are easy to use, they have been shown to have a better responsiveness (i.e., ability to detect significant changes), and validity (i.e., the ability to assess more closely the actual experience of participants) as compared to other instruments such as Likert scales [49–51]. The experimental set-up is illustrated in Fig 1.

#### 2.3.3 Physiological arousal

Electrodermal Activity (EDA) signals (i.e., the electrical activity of the skin resulting from changes in sweating) were recorded using the Biopac MP150 Systems (Version 4.2, Biopac Systems Inc., CA, USA), in three time windows: during the first repetition of the SSDT (Pre), during the touch manipulation, and during the second repetition of the SSDT (Post). The three recordings were interspersed with 90s breaks. Two electrodes were placed on the index and the middle finger of the left hand. The electrodes were connected to the Biopac MP150 Systems and the Biopac Student Lab Pro 3.7 software, which was programmed to filter in real time EDA data with a band-pass of 0–35Hz. The sampling rate for data acquisition was set at 1000Hz.

EDA data were then used for calculating Skin Conductance Levels (SCLs), a measure of the background tonic EDA that is commonly used as an index of psychological arousal. Particularly, SCLs are deemed to reflect slow changes in the autonomic sympathetic nervous system (SNS) in response to stressors or emotional stimuli [52]. Higher SCLs indicate a stronger activation of the SNS and therefore a higher level of arousal [36]. In the current study, SCLs were preferred over cardiac measures of physiological arousal, in accordance with previous research that suggested EDA to be a better index for detecting arousal changes when investigating responses that are predominantly of lower arousal [36, 52]. However, tactile stimuli have been previously reported to affect also other autonomic and cardiac measures (e.g., [53–55]). SCLs were recorded to investigate changes in arousal due to the touch manipulation, and to analyse possible influences of arousal levels on subsequent SSDT responses.

### 2.4 Self-report questionnaires

Self-report measures were used to control the sample for possible confounding variables that have been shown to impact touch perception during the SSDT, that are eating disorder symptoms, body dysmorphic disorder symptoms and individual differences in body awareness [29, 56, 57]. Pre-existing abnormalities and between groups differences in these variables could indeed bias interpretation of results. Scores were compared to normative data to ensure comparability with the general population, and were compared statistically between the two groups to ensure homogeneity of participants.

#### 2.4.1 Eating Disorder Inventory-3 (EDI-3; [58])

The EDI-3 is 91-item self-report questionnaire used to assess disordered eating symptomatology. Participants are asked to rate how frequently they do what is reported in each item on a 6-point Likert scale ranging from “never” to “always”. Example items are: “I eat when I’m upset”, “I stuff myself with food”, “I think about dieting” and “I think my hips are too big”. The EDI-3 comprises 12 subscales assessing different domains defining, and/or associated with EDs: Drive for Thinness, Bulimia, Body Dissatisfaction, Low Self-Esteem, Personal Alienation, Interpersonal Insecurity, Interpersonal Alienation, Interoceptive Deficits, Emotional Dysregulation, Perfectionism, Asceticism, and Maturity Fears. In the current study, we focused on the ED Risk Composite which constitutes an index of the risk to develop an ED and correspond to the sum of scores on the Drive for Thinness, Bulimia and Body Dissatisfaction subscales. Clinical samples have been found to score a mean value of 61 on the ED Risk Composite (between a minimum of 51 and a maximum of 75; [59]). The EDI-3 has been found to have an excellent specificity and sensitivity, and a good internal consistency (α  =  between .75 and .92; [59]).

#### 2.4.2 Dysmorphic Concerns Questionnaire (DCQ; [60])

The DCQ is a 7-item self-report scale investigating body image preoccupation and dysmorphic concerns. It includes items such as: “Have you ever been very concerned about some aspect of your appearance?” and “Have you ever spent a lot of time worrying about a defect in your appearance or bodily functioning?”. Items are rated on 4-point Likert scale from a minimum of 0 (“not at all”) to a maximum of 4 (“much more than most people”). Previous research indicated that total scores above 9 are indicative of clinical concern [61]. The DCQ was shown to have a good internal consistency with α  =  .80 [62].

#### 2.4.3 Body Perception Questionnaire-Very Short Form (BPQ-VSF; [63])

The BPQ is a 12-item self-report questionnaire of body awareness and autonomic reactivity. Participants are asked to state how often they consider themselves to be aware of different bodily signals such as: “muscle tension”, “goose bumps”, “digestive problems”, and “heart-beating”. Items are rated on a 5-point Likert scale ranging from 1 (“Never”) to 5 (“Always”). Total scores range between 12 and 60, with higher values reflecting a hypersensitivity to bodily sensations, lower values a hyposensitivity, and average scores a normal sensitivity. The BPQ has been shown to have an excellent test-retest reliability and a good internal consistency (α  =  between .83 and .91; [64]).

### 2.5 Procedure

#### 2.5.1 General procedure

At the beginning of each testing session, the experimenter took a photograph of the participant’s face using a Nikon D50 digital SLR camera. Participants were standing against a plain-coloured grey background, and they were photographed with a neutral facial expression, without hair covering their face. Each participants’ original photograph was centred and cropped to adjust to the computer screen, and it was flipped horizontally as seen in a mirror using Microsoft Picture Manager 2013. The photograph of the participant was presented on the computer monitor during all trials of SSDT as part of the set-up (see above). Subsequently, electrodes were placed on participants’ hand as previously explained for recording EDA.

**Fig 2:**
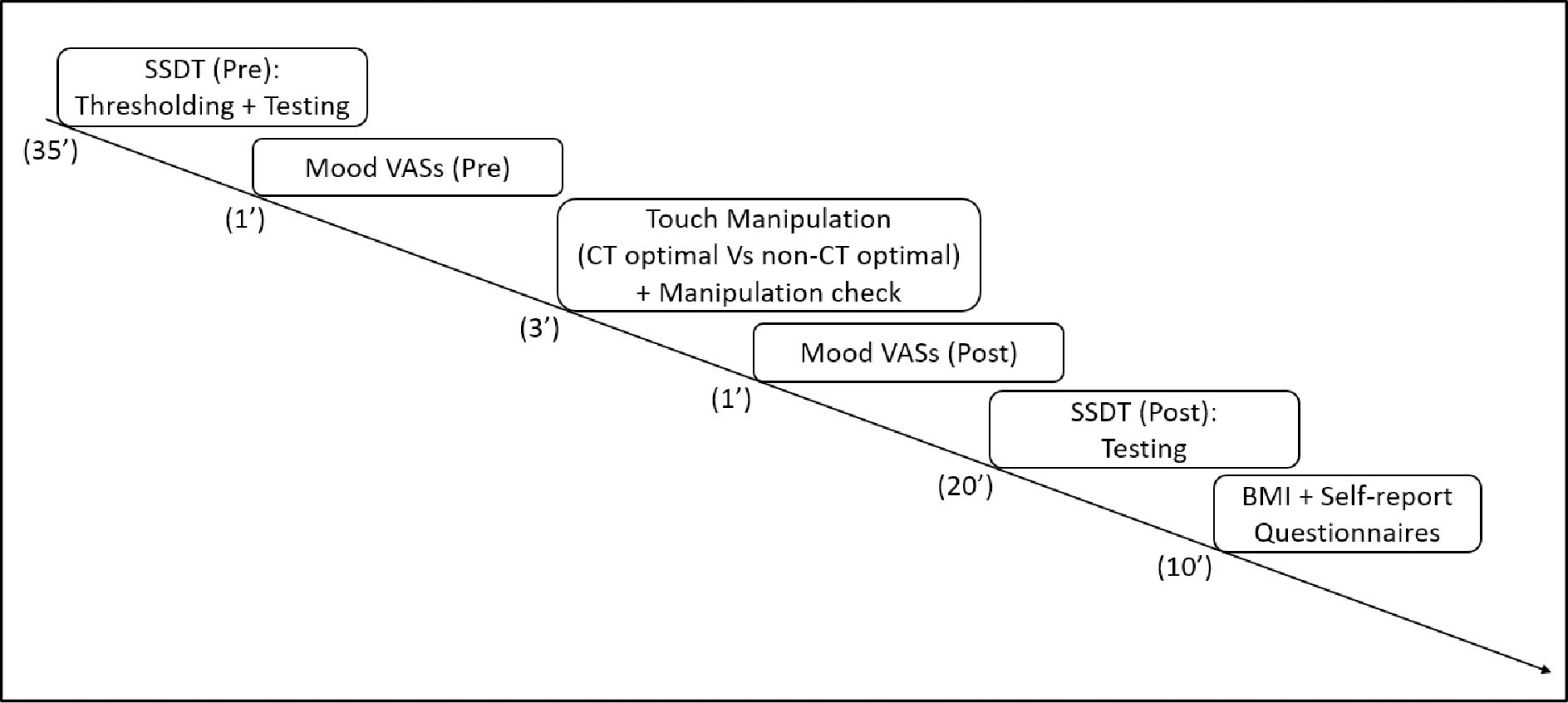
Timeline representation of the different steps of the study procedure.

Participants were then asked to complete the first repetition of the SSDT protocol (Pre), which consisted of a thresholding procedure and a testing phase (see below). After completing the SSDT, participants were asked to fill in the mood VASs (Pre), they were administered with the touch manipulation according to their group assignment (CT optimal vs non-CT optimal), and they were then asked to fill in the mood VASs a second time (Post). After the touch manipulation, participants repeated the testing phase of the SSDT a second time (Post). They were then administered each of the self-report questionnaires: the EHI, the EDI-3, the DCQ and the BPQ. Lastly, height and weight were measured with a stadiometer and a scale for calculating the Body Mass Index (BMI; kg/m^2^) using the NHS online calculator. The testing procedure lasted approximately 90 minutes. A schematic representation of the general procedure is presented in Fig 2.

#### 2.5.2 SSDT thresholding procedure

Replicating the methods used by Mirams and colleagues [65], before the beginning of the testing phase, participants completed a thresholding procedure to individually calibrate the strength (amplitude) of the tactile pulse used during the subsequent testing phase. A threshold was found for each participant using the Parameter Estimation by Sequential Testing (PEST; [66]) algorithm. Participants were presented with a series of pairs of trials (1,020ms each), during which either in the first or in the second trial a 20ms tactile pulse (Touch) was delivered with a delay of 500ms on either side (500ms–Touch– 500ms). The other trial consisted of an empty 1,020ms period during which no touch occurred (No Touch). The beginning of each trial was signalled by a 250ms beep sound administered through a pair of earphones. Participants were then asked to decide whether they felt a pulse during the first or second trail in a two alternative forced choice design (i.e., “Did you feel the vibration during the first or second trial?”) by pressing “1” or “2” on the computer keyboard.

The PEST procedure was set to identify the intensity necessary for participants to detect the pulse in 75% of trials (75% threshold; [65]). The procedure began by presenting the same above threshold tactile pulse to all participants. If participants responded correctly on a series of trials (> 75% correct responses), the programme automatically reduced the strength of the tactile pulse. Conversely, if they began to respond incorrectly (< 75% correct), the programme automatically increased the strength of the pulse. A Wald [67] sequential likelihood-ratio test was used to determine when to change the strength of the stimulus. The thresholding procedure took approximately 15 minutes. If the minimum step size was not reached after 120 trials, the strength of the tactile pulse was set to the average stimulus strength over the last 50 thresholding trials. Immediately after completing the thresholding procedure, participants started the experimental phase of the SSDT.

The thresholding procedure was performed only once during the first repetition of the SSDT. The strength of the tactile pulse was then set at the same threshold level during the first and the second repetitions of the testing phase of the SSDT.

#### 2.5.3 SSDT testing phase

At the beginning of the testing phase, the experimenter placed the LED on the computer monitor on the right cheek of the photograph presented, therefore mirroring the position of the tactor on participants’ face. The testing phase of the SSDT employed a repeated-measures design with tactile pulse (Touch/No Touch) and light (Light/No Light) as within-subjects factors. The tactile pulse was administered in 50% of trials. Simultaneously, the LED flashed in 50% of trials, giving the following four trial types: touch only (No Light/Touch); light only (Light/No Touch); light and touch (Light/Touch); and catch (No Light/No Touch). Each trial type was presented 20 times in a random order, giving a total number of 80 trials. As in the thresholding procedure, the beginning of each trial was signalled by an auditory cue followed by a 1,020ms stimulus window. As during the thresholding procedure, in touch only trials, a 20ms tactile pulse was administered alone preceded and followed by a 500ms delay. In light and touch trials, the LED flashed for 20ms synchronously with the tactile pulse. In light only trials, the LED flashed for 20ms alone. In catch trials, no stimulation was administered. At the end of each trial, participants were asked to report whether or not they felt a touch (i.e., “Did you feel a vibration?”), and they were given four possible answers: ‘definitely yes’ (keyboard button ‘1’), ‘maybe yes’ (keyboard button ‘2’), ‘maybe no’ (keyboard button ‘3’), or ‘definitely no’ (keyboard button ‘4’; [68]). For the purposes of this study and consistent with previous studies using the SSDT, ‘definitely’ and ‘maybe’ responses were combined in a yes/no binary coding [29, 30, 68].

## 3. Results

### 3.1 Data processing

#### 3.1.1 SSDT outcomes

Participants’ responses on the SSDT were classified as hits (reports of feeling the touch on touch-present trials), false alarms (erroneous reports of feeling the touch on touch-absent trials), misses (reports of not feeling the touch on touch-present trials), or correct rejections (reports of not feeling the touch on touch-absent trials; [28]). According to the log-linear correction, hit rates (HR) were calculated using the formula: [hits + .5/(hits + misses +1)], and false alarm rates (FA) using the formula: [false alarms + .5/(false alarms + correct rejections +1)] [69]. HR and FA were then used to calculate the signal detection theory test statistics *d*′ and *c*, where *d*′ (i.e., sensitivity) indicates participants’ ability to discriminate between touch-present and touch-absent trials [zHR − *z*FA], and *c* (response criterion) indicates participants’ tendency to report stimuli as present regardless of the type of trail [−.5*zHR + *z*FA] [70]. Lower scores on *c* (*c* < 0) indicate a higher tendency to report touch (answer “yes”) across trials.

#### 3.1.2 SCLs data extrapolation

EDA data were recorded continuously during three time windows: the first repetition of the SSDT (SSDT Pre), the touch manipulation, and the second repetition of the SSDT (SSDT Post), resulting on a total of 3 recordings per participant (each lasting approximately 15 minutes; see Fig 2). Each recording was visually inspected for artefacts which were manually removed using Biopac (MP150) Systems. Three SCLs per participant were then extrapolated averaging across the EDA signal in each recording.

### 3.2 Demographics and self-reports analyses

Statistical analyses were performed using SPSS (SPPS Inc., Chicago, IL). All data are reported as Mean (*M*) and Standard Deviation (*SD*). A significance threshold of *p* < .05 was set for all effects, and effect sizes were estimated using Cohen’s *d* and partial eta square (*η*^2^).

A series of t-tests was performed to analyse group differences in demographics, baseline mood, and self-report personality traits. As reported in Table 1, the two groups (CT vs non-CT) were found not to differ in Age, SSDT threshold levels, or Body Mass Index (BMI). No significant between-groups differences were found in mood prior to the touch manipulation, nor in eating disorder symptoms (EDI), body dysmorphic symptoms (DCQ), or body awareness (BPQ). The two groups were therefore comparable in all measures that were identified as possible confounding variables. Moreover, mean scores for the EDI, DCQ, and BPQ of our two groups were consistent with mean scores of the normative healthy samples, indicating our sample to be representative of the general population [59, 61, 64].

**Table 1:** Descriptive statistics for Age, SSDT threshold levels, mood at baseline and questionnaire scores in each group.

	CT optimal	non-CT optimal				
	M (sd)	M (sd)	t	df	sig	d
Age	32.09 (15.32)	31.97 (12.75)	.12	64	.90	.03
Threshold	-1067.31 (390.86)	-1085 (394.27)	.18	64	.85	.04
BMI	26.37 (5.81)	25.18 (5.56)	.85	64	.40	.21
Calmness_Pre	79.82 (19.66)	72.94 (20.55)	1.39	64	.17	.34
Anxiety_Pre	24.89 (24.09)	25.55 (23.48)	-.11	64	.91	.03
Happiness_Pre	70.49 (16.53)	72.62 (16.07)	-.53	64	.60	.13
Sadness_Pre	17.23 (19.22)	15.97 (19.47)	.28	64	.78	.06
EDI-3	32.19 (18.25)	29.47 (21.31)	.55	64	.58	.14
DCQ	6.56 (3.93)	6.12 (4.14)	.44	64	.66	.11
BPQ	35.94 (12.35)	36.50 (11.69)	-.190	64	.85	.05

### 3.3 SSDT analyses

Descriptive statistics for HR, FA, *d′* and *c* in each Light and Time condition of the SSDT, in each group are presented in Table 2. Before performing the analyses, these SSDT outcomes were tested for normality. HR, *d′* and *c* were normally distributed, therefore parametric analyses were conducted. Based on the main hypothesis of the study that the touch manipulation would increase *d’* in the CT optimal group only, independent sample t test were planned a-priori to compare *d’* in the CT vs non-CT optimal group before and after the touch manipulation. Three repeated-measures mixed design ANOVAs were then performed with Group as the between-subject factor, and Light and Time as within-subject factors, using HR, *d’* and *c* as dependent variables. Paired and independent-sample t tests were performed to follow up significant interactions. Bonferroni correction was used to correct for multiple comparisons.

**Table 2:** Descriptive statistics for hit rate, false alarm rate, d’ and c in each Light (Light and No Light) and Time (Pre and Post) condition during the SSDT, in each group (CT and non-CT).

CT optimal		HR (%)	FA (%)	*d’*	*c*
		M (SD)	M (SD)	M (SD)	M (SD)
Pre	No Light	47.69 (21.45)	10.55 (6.97)	1.41 (.77)	.78 (.43)
	Light	59.53 (25.28)	8.43 (7.09)	1.58 (.91)	.45 (.43)
Post	No Light	44.69 (27.94)	7.74 (7.45)	1.41 (1.03)	.92 (.50)
	Light	53.34 (26.97)	6.46 (6.55)	1.66 (1.03)	.73 (.53)
Non-CT optimal		HR (%)	FA (%)	*d’*	*c*
		M (SD)	M (SD)	M (SD)	M (SD)
Pre	No Light	44.30 (26.68)	9.44 (7.10)	1.35 (.90)	.89 (.52)
	Light	46.91 (25.24)	7.74 (6.93)	1.30 (.82)	.77 (.48)
Post	No Light	33.50 (23.14)	9.21 (7.21)	.91 (.69)	.97 (.56)
	Light	45.82 (30.14)	8.59 (6.44)	1.19 (.93)	.83 (.57)

Conversely, FA in all experimental conditions were not normally distributed, with a significant positive skewness. As FA remained not normal after attempts to transform the data, non-parametric analyses were conducted. As there is no non-parametric test equivalent to a mixed design ANOVA, two Freidman’s ANOVAs, for the CT optimal and the non-CT optimal groups, were used to investigate within-subjects effects, with Light and Time as within-subject factors. Wilcoxon tests were used to follow up significant results. Mann-Whitney U tests were used to investigate between-subject effects.

#### 3.3.1 Hit Rate (HR)

There was a significant main effect of the Light (*F*(1,64) = 33.48, *p* = .000, *η*^2^ = .34) and a significant three way interaction Time × Light × Group (*F*(1,64) = 5.73, *p* = .020, *η*^2^ = .08). Follow-up analyses showed higher HR in Light compared to No Light trials in the CT optimal group (*F*(1,31) = 19.98, *p* = .000, *η*^2^ = .39; Light: *M* = 56.44, *SD* = 21.90; No Light: *M* = 46.19, *SD* = 21.80). Conversely, in the non-CT optimal group there was no main effect of the Light, driven by the fact that before the touch manipulation the presence of the light did not increase HR (Pre; *t*(33) = -1.05, *p* = .30, *d* = .18). This indicates that during the first repetition of the SSDT (Pre), for the non-CT optimal group, HRs were less influenced by the presence of the Light than the CT optimal group.

Indeed, in the non-CT optimal group the effect of the Light was present only after the touch manipulation (Post; *t*(33) = -3.89, *p* = .000, *d* = .67) showing higher HR in Light (*M* = 45.82, *SD* = 30.14) compared to No Light (*M* = 33.50, *SD* = 23.14) trials. However, this effect seemed to be driven by the fact that HR dropped down significantly in No Light trials from before (Pre: *M* = 44.30, *SD* = 26.68) to after the touch manipulation (Post: *M* = 33.50, *SD* = 23.14; *t*(33) = 2.89, *p* = .007, *d* = .50), indicating that participants in the non-CT optimal group were less able to perceive the touch after the touch manipulation without the prompt of the Light. No main effect of Time and Group, and no interactions were found to be significant (all *Fs* ≤ 1.98; all *ps* ≥ .16).

**Fig 3:**
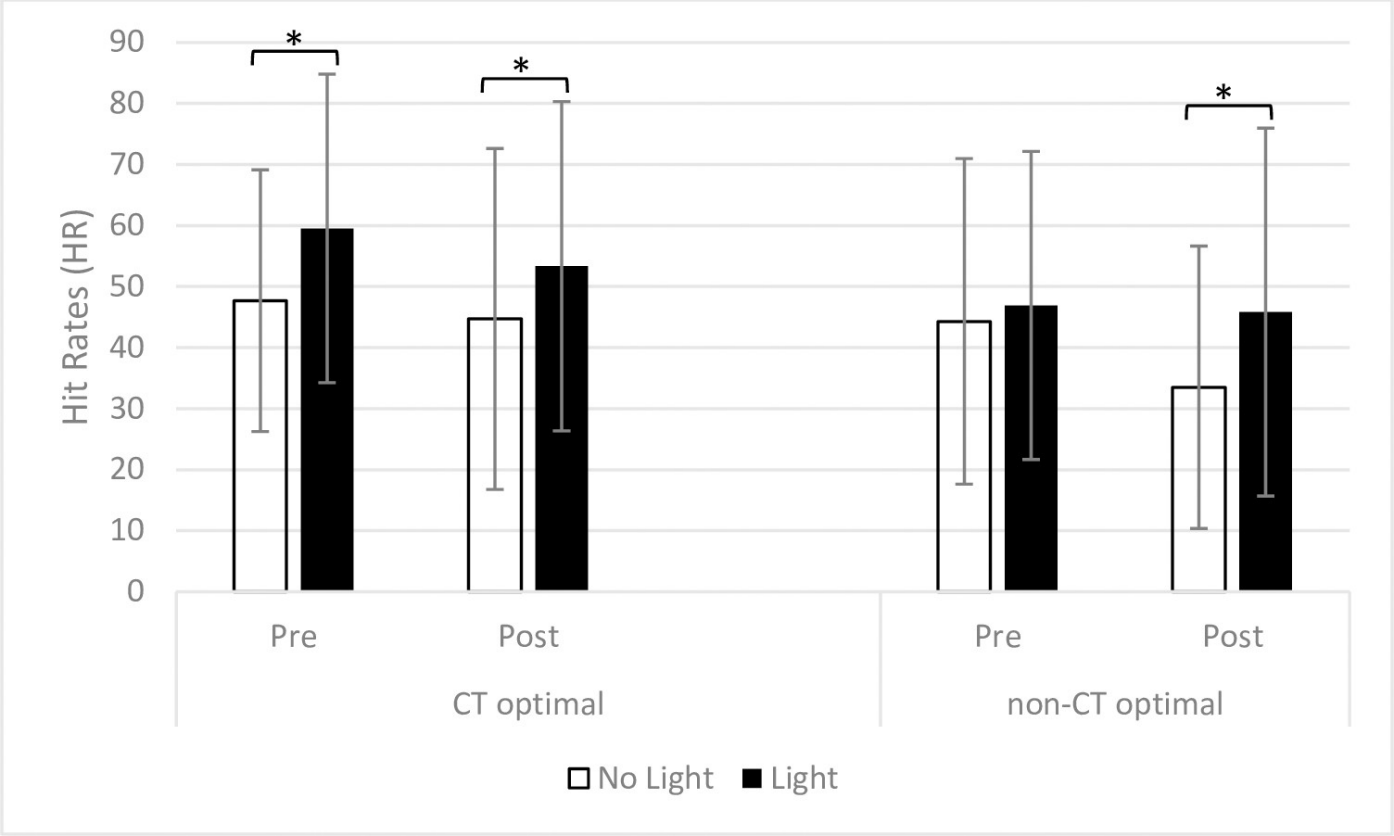
Mean HR during the SSDT in Light and No Light trials before (Pre) and after (Post) receiving the touch manipulation in the CT optimal and non-CT optimal group. Error bars show standard deviations. For the CT optimal group there was an effect of the light during the first (Pre) and the second repetition (Post) of the SSDT. For the non-CT optimal group an effect of the light was found only during the second repetition (Post).

Analyses were repeated using the difference score in HR from the first (baseline) and the second repetition of the SSDT to correct for between-group differences at baseline. Results showed a significant Light × Group interaction (*F*(1,64) = 19.98, *p* = .020, *η*^2^ = .08). Post-hoc analyses showed an effect of the Light in the non-CT optimal group only (*t*(33) = -.09, *p* = .020, *d* = .23). This is consistent with previous results showing in this group an effect of the light only after the touch manipulation. Therefore the correction for baseline scores did not change this effect. No significant results were found in the CT optimal group resulting from the fact that the effect of the Light before and after the touch manipulation remained fairly stable. No other significant results were found (all *ts* ≤ 1.38; all *ps* ≥ .17). Results are reported in Fig 3.

#### 3.3.2 False Alarms (FA)

A Friedman’s test showed that there was a significant difference in FA in at least one of the four experimental conditions (Light × Time) in the CT optimal group (X^**2**^(3) = 19.32, *p* = .000, *W* = .20). Post-hoc tests using Wilcoxon signed-rank test showed that in the CT optimal group there was a significant effect of Time with lower FA after the touch manipulation (Post; *Mdn* = 4.50) as compared to before it (Pre; *Mdn* = 8.90; *z* = -2.52, *p* = .012, *r* = .45), indicating that receiving CT optimal touch decreased subsequent false reports of the tactile pulse during the SSDT. No other significant within-subjects effects were found in the CT optimal group (all *zs* ≤ -1.85, all *ps* ≥ .064). Regarding the non-CT optimal group, a Friedman’s test showed that there was a tendency towards a significant difference in FA between the four experimental conditions (Light × Time; X^**2**^(3) = 7.58, *p* = .056, *W* = .07). However, follow up analyses showed no within-subjects effects to be significant (all *zs* ≤ -1.86, all *ps* ≥ .083).

**Fig 4:**
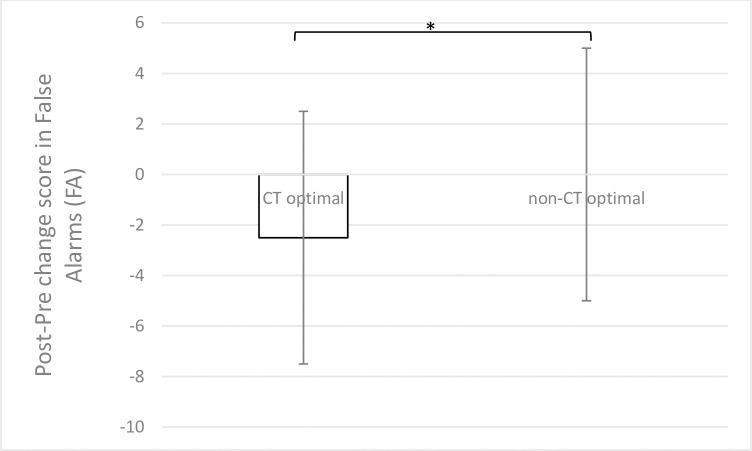
Median change score in FA between the first (Pre) and the second (Post) repetition of the SSDT in the CT optimal and non-CT optimal group. Error bars show interquartile ranges (IQR). The CT optimal group showed a stronger decrease in FA during the second repetition (Post) of the SSDT as compared to the non-CT optimal group.

Mann-Whitney tests revealed a significant between-groups difference in FA in the change score between Pre and Post FA (before and after the touch manipulation; *U* = 376.50, *z* = -2.18, *p* = .029, *η*^2^ = .07), with a stronger decrease in FA from Pre to Post in the CT optimal group (*Mdn* = -2.50) as compared to the non-CT optimal group (*Mdn* = .00). No other significant differences between the two groups were found (*U* ≤ 406; *p* ≥ .062). Results are reported in Fig 4.

#### 3.3.3 Sensitivity (*d’*)

**Fig 5:**
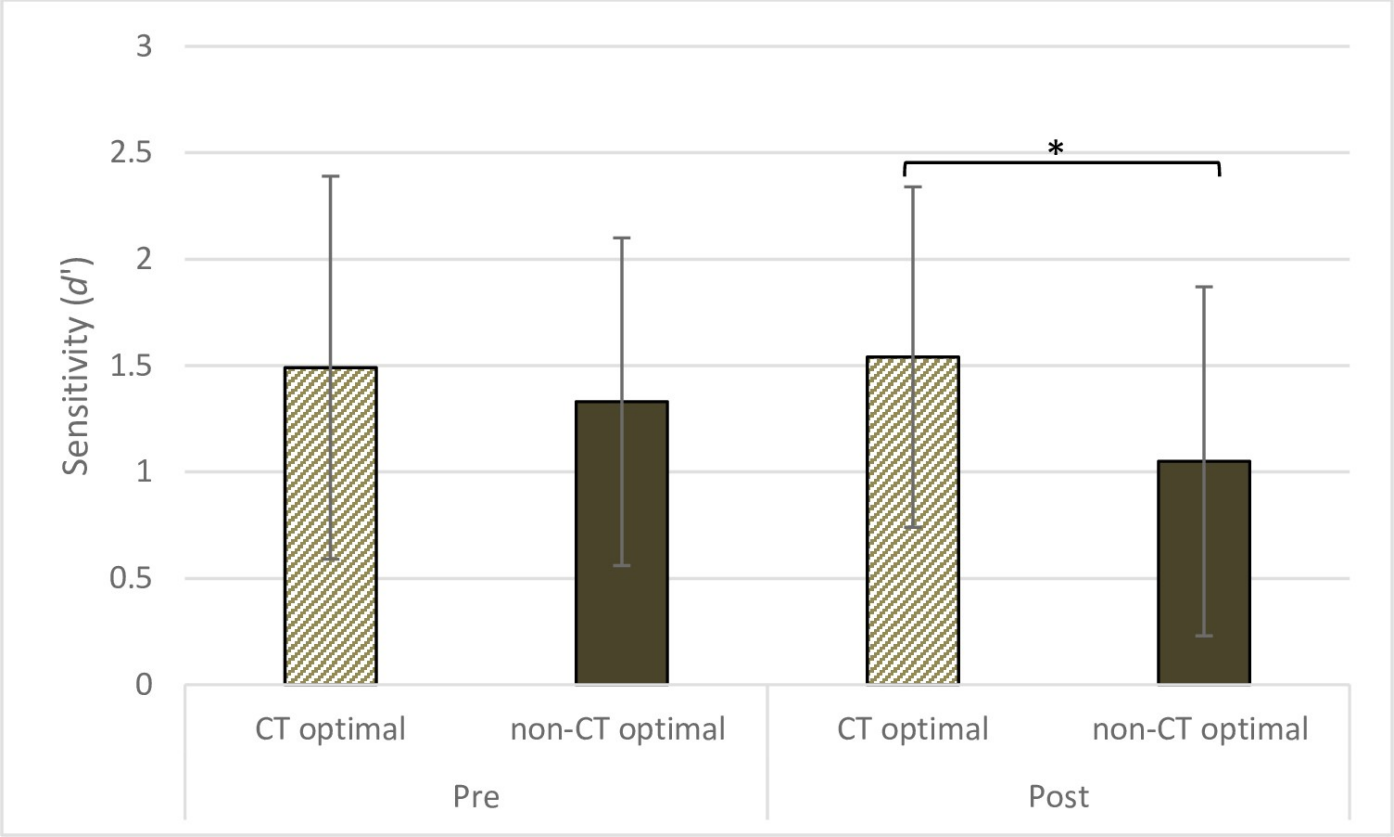
Mean *d’* during the SSDT before (Pre) and after (Post) receiving the touch manipulation in the CT optimal and non-CT optimal group. Error bars show standard deviations. the CT optimal group showed a significantly higher *d’* compared to the non-CT optimal group only during the second repetition (Post) of the SSDT.

Following the main hypothesis of this study, a priori analyses were planned to assess differences in *d’* between the CT optimal and the non-CT optimal groups before (Pre) and after (Post) the touch manipulation. According to our expectations, results showed a significantly higher *d’* in the CT optimal vs the non-CT optimal group only after the touch manipulation (Post; *t*(64) = 2.36, *p* = .022, *d* = .57; CT optimal: *M* = 1.54, *SD* = .90; non-CT optimal: *M* = 1.05, *SD* = .77, see Fig 5). This difference may reflect the fact that a decrease in FA after the touch manipulation was found in the CT optimal group only. Conversely, there was no between-groups difference in *d’* before the touch manipulation (Pre; *t*(64) = .84, *p* = .403, *d* = .21).

In order to have a more thorough description of the data and consistently with the analyses run for the other SSDT outcomes, a repeated-measures mixed design ANOVA was also performed. Results showed a main effect of Light with higher *d’* in Light (*M* = 1.43, *SD* = .80) compared to No Light (*M* = 1.27, *SD* = .74) trials. Moreover, there was a tendency towards a main effect of Group (*F*(1,63) = 2.97, *p* = .071 *η*^2^ = .04) due to a higher *d’* in the CT optimal (*M* = 1.51, *SD* = .73) compared to the non-CT optimal group (*M* = 1.19, *SD* = .71). This tendency was probably driven by the fact that the CT optimal showed a higher *d’* after the touch manipulation (Post) as explained before.

There was no significant main effect of Time, and no two or three way interactions were significant (all *Fs* ≤ 1.98; all *ps* ≥ .16). Therefore, it should be noted that there was no significant Group × Time interaction to support the analyses of the a priori simple effects comparing *d’* in the CT optimal vs the non-CT optimal groups before (Pre) and after (Post) the touch manipulation. Although, as argued by Howell [71], significant interactions in the omnibus ANOVA are unnecessary for performing simple effects analyses when specific simple effects are predicted a priori by the study’s hypothesis, we also believe that this non-significant interaction should be acknowledged as a limitation of current results.

#### 3.3.4 Response criterion (*c*)

There was a significant main effect of Time (*F*(1,64) = 7.71, *p* = .007, *η*^2^ = .10) with higher *c* during the second repetition of the SSDT (Post; *M* = .87, *SD* = .50) compared to the first repetition of the SSDT (Pre; *M* = .73, *SD* = .46), indicating that participants were overall more inclined to report feeling the tactile pulse, regardless whether it was administered or not, before (Pre) rather than after (Post) the touch manipulation.

There was also a main effect of the Light (*F*(1,64) = 37.75, *p* = .000, *η*^2^ = .37) and a significant Light × Group interaction (*F*(1,64) = 4.65, *p* = .035, *η*^2^ = .07). Follow-up analyses showed a lower *c* in Light compared to No Light trials in both the CT optimal (*t*(31) = 6.88, *p* = .000, *d* = 1.21; Light: *M* = .59, *SD* = .41; No Light: *M* = .85, *SD* = .42) and the non-CT optimal group (*t*(33) = 2.53, *p* = .016, *d* = .43; Light: *M* = .80, *SD* = .46; No Light: *M* = .93, *SD* = .47). Further follow up analyses showed no other significant between-groups differences in *c*. However, when looking at change scores between Light and No Light trials, the CT optimal group showed a stronger decrease in *c* from No Light to Light trials (*M* = -.26, *SD* = .22) compared to the non-CT optimal group (*M* = -.13, *SD* = .29; *t*(64) = -2.16, *p* = .035, *d* = .53). However this last difference was no longer significant when using Bonferroni correction. Overall, these results indicate that all participants had a higher tendency to report perceiving the tactile pulse when the Light was present; however, this tendency was slightly stronger in the CT optimal group (possibly due to the fact that the non-CT optimal group showed no effect of the Light on HR before the touch manipulation; see below for a summary).

There was no significant main effect of Group, and no other two or three way interactions were significant (all *Fs* ≤ 1.98; all *ps* ≥ .16).

#### 3.3.5 SSDT results summary

Overall SSDT outcomes showed that in the CT optimal group, according to previous literature, the presence of the light induced an increase in HR as compared to trials in which the light was absent [29, 30]. Conversely, in the non-CT optimal group the presence of the light was found to induce higher HR only after receiving the touch manipulation. However, this effect was driven by the fact that after receiving the non-affective touch, there was a decrease in HR in trials during which the light was absent. This suggests that participants in the non-CT optimal group were less able to perceive the touch after the touch manipulation when there was not the prompt of the light.

Conversely, receiving affective touch was found to decrease FA, with participants in the CT optimal group reporting less FA after the touch manipulation. Accordingly, the change score in FA from before to after the touch manipulation was significantly greater in the CT optimal group as compared to the non-CT optimal group. Results in FA were partially mirrored by results in *d’*. Indeed, the CT optimal group showed a tendency toward a higher *d’* after the touch manipulation when compared to the non-CT optimal group. Taken together, results in FA and *d`*suggest that affective touch induced an increase in tactile accuracy. However, analyses in *d’* were exploratory and followed-up a main effect of Group that was only approaching significance and as such they should be interpreted with cation.

Moreover, there were significant effects of the Light on *d’* and *c* indicating that the presence of the light induced participants to correctly report perceiving the tactile pulse more often. This effect of the Light on *c* was slightly stronger in the CT optimal group, which exhibited a higher change score between Light and No Light trials compared to the non-CT optimal group. However, this difference in *c* between the two groups seemed to be driven by previously shown results in HR, where the presence of the light was found to induce an increase in HR in the CT optimal group only. In contrast to some previous studies, there was no effect of the Light on FAs [28, 68].

### 3.4 CT vs non CT touch manipulation check

Two Independent sample t-tests were performed with Intensity and Pleasantness as dependent variables and Group as a between-subjects factor. According to our expectations and in accordance with previous literature, the CT optimal group rated the touch manipulation as significantly more pleasant (*M* = 6.72, *SD* = 3.09; *t*(64) = 5.73, *p* = .000, *d* = 1.42) and less intense (*M* = 33.44, *SD* = 23.16; *t*(64) = -3.46, *p* = .001, *d* = .85) compared to the non-CT optimal group (Pleasantness: *M* = 1.70, *SD* = 3.94; Intensity: *M* = 51.09, *SD* = 18.05; [7, 8, 48].

### 3.5 Mood VASs

Four mixed design ANOVAs were performed with Time as within-subject factor and Group as a between-subjects factor to assess how the touch manipulation effected mood (Pre vs Post the touch manipulation) in the CT optimal vs non-CT optimal group. The touch manipulation increased participants’ self-reported Calmness (*F*(1,64) = 4.36, *p* = .041, *η*^2^ = .06; Pre: *M* = 76.28, *SD* = 20.27; Post: *M* = 81.01, *SD* = 17.76) and Happiness (*F*(1,64) = 7.65, *p* = .007, *η*^2^ = .11; Pre: *M* = 71.59, *SD* = 16.20; Post: *M* = 76.17, *SD* = 16.19), and decreased participants’ self-reported Anxiety (*F*(1,64) = 12.56, *p* = .001, *η*^2^ = .16; Pre: *M* = 25.23, *SD* = 23.60; Post: *M* = 16.74, *SD* = 20.06) and Sadness (*F*(1,63) = 5.36, *p* = .018, *η*^2^ = .09; Pre: *M* = 16.59, *SD* = 19.21; Post: *M* = 12.96, *SD* = 17.05). However, contrary to our expectations, these effects were not specific for the CT optimal group. Indeed, there were no significant main effects of Group and no Time × Group interactions were significant (all *Fs* ≤ 1.91; all *ps* ≥ .17).

### 3.6 Physiological arousal

**Fig 6:**
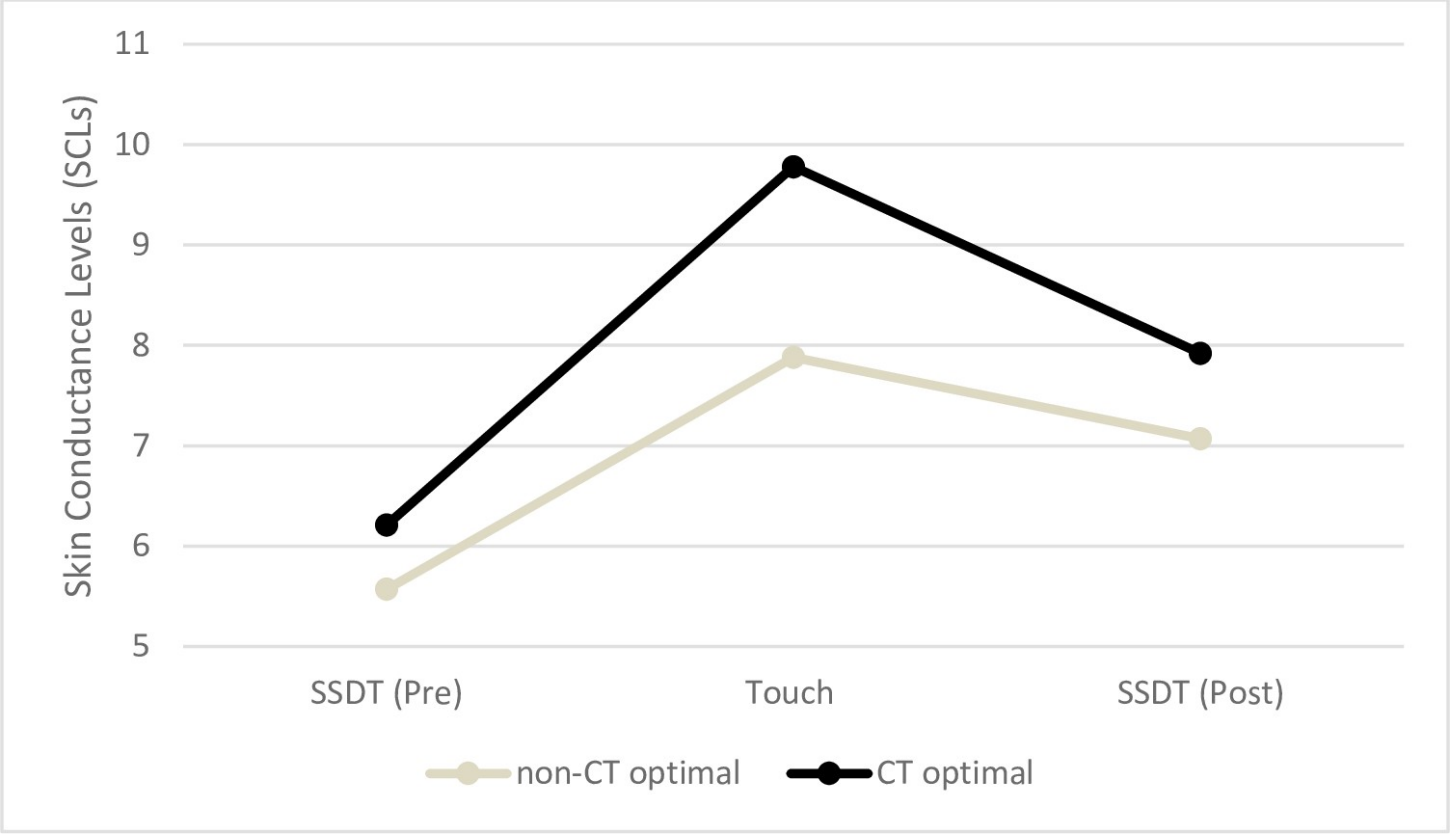
SCLs in the CT optimal and in the non-CT optimal groups during the first repetition of the SSDT (SSDT Pre), the touch manipulation (Touch), and the second repetition of the SSDT (SSDT Post). SCLs increased during the touch manipulation, especially for the CT optimal group.

A mixed ANOVA was performed with Time as a within-subjects factor, Group as a between-subjects factor and SCLs as the dependent variable, to analyse changes in physiological arousal during testing. For this analysis three temporal windows were considered: the first repetition of the SSDT (SSDT Pre), the touch manipulation (Touch) and the second repetition of the SSDT (SSDT Post). Therefore, in the context of the current analyses the factor Time had three levels. Results showed a significant main effect of Time (*F*(2,64) = 59.67, *p* = .000, *η*^2^ = .48) and a significant Time × Group interaction (*F*(2,64) = 3.13, *p* = .047, *η*^2^ = .05). Follow-up analyses indicated that SCLs during Touch (*M* = 8.87, *SD* = 4.79) were significantly higher compared to both the SSDT Pre (*M* = 6.04, *SD* = 4.46; *t*(65) = -10.69, *p* = .000, *d* = .61) and the SSDT Post time window (*M* = 7.53, *SD* = 5; *t*(65) = 5.30, *p* = .000, *d* = .27). Moreover, SCLs during the SSDT Post time window were found to be significantly higher than SCLs during the SSDT Pre time window (*t*(65) = -10.69, *p* = .000, *d* = .61). Further Independent sample t-tests indicated that the CT optimal group had a significantly higher change score in SCLs from SSDT Pre to Touch (*M* = 3.47, *SD* = 2.32) as compared to the non-CT optimal group (*M* = 2.22, *SD* = 1.80; *t*(64) = 2.45, *p* = .017, *d* = .60). No other between-groups comparisons were found to be significant (all *ts* ≤ 1.40; all *ps* ≥ .17). Overall results indicated that the touch manipulation induced an increase in arousal that was slightly higher in the CT optimal group as compared to the non-CT optimal group. After the touch manipulation, during the second repetition of the SSDT, the level of arousal decreased. However, it remained significantly higher than during the first repetition of the SSDT. SCLs data are presented in Fig 6.

### 3.7 Exploratory correlational analyses

No a priori hypotheses drove these analyses, they were rather run for exploratory reasons and with the aim to inform future research in this field.

#### 3.7.1 SSDT outcomes, mood and arousal

Exploratory Pearson’s and Spearman’s correlations were run to test whether participants’ emotional states (Mood VASs), levels of arousal (SCLs), and perceived quality of touch (Intensity and Pleasantness) were related to performance during the SSDT (HR, FA, *d’* and *c* mean values across all experimental conditions). There was a relationship between the HR and *d’* indices and levels of arousal (SCLs). Particularly, there was a significant positive correlation between overall HR (across all experimental conditions) and SCLs during the touch manipulation (*r* = .34; *p* = .005) and SCLs during the second repetition of the SSDT (Post; *r* = .28; *p* = .023). However, this last correlation did not remain significant when using a Bonferroni correction. Alongside *d’* (across all experimental conditions) was positively correlated to SCLs in all three temporal windows: during the first repetition of the SSDT (*r* = .27; *p* = .027; only approaching significance when using a Bonferroni correction), the touch manipulation (*r* = .38; *p* = .002) and the second repetition of the SSDT (*r* = .32; *p* = .009). Overall, these results suggest that participants exhibiting higher levels of arousal were also more accurate in correctly reporting the tactile pulse during the SSDT as indicated by HR and *d’*. Conversely, SSDT responses were not related to participants’ emotional states (as measured by the Mood VASs), or perceived quality of touch (ratings of Intensity and Pleasantness; all *rs* ≤ .17; all *ps* ≥ .18).

#### 3.7.2 Manipulation check, mood and arousal

Further correlational analyses were run to analyse the relationship between participants’ perceived quality of touch during the touch manipulation (Intensity and Pleasantness; i.e., manipulation check), and subsequent emotional states (Mood VASs_Post) and levels of arousal. Perceived Pleasantness of touch was found to be positively correlate with subsequent Calmness (*r* = .35; *p* = .004) and negatively correlated with subsequent Anxiety (*r* = .34; *p* = .005). Therefore, the more pleasant the touch manipulation was perceived the calmer and less anxious participants felt after it. Moreover, Intensity of touch was found to be positively correlated with subsequent Sadness (*r* = .28; *p* = .020), suggesting that the more intense the touch was perceived the sadder participants were after the touch. However, this correlation did not remain significant when using a Bonferroni correction. There were no other significant correlations (all *rs* ≤ -.17; all *ps* ≥ .16).

## 4. Discussion

The aim of the current study was to investigate the off-line carry-over effects of CT optimal (affective) vs non-CT optimal (non-affective) touch on subsequent visuo-tactile multisensory integration using the Somatic Signal Detection Task (SSDT). According to previous literature, linking CT optimal affective touch to body awareness and multisensory perception, we predicted affective touch to enhance perceptual accuracy during the subsequent SSDT as compared to non-affective touch.

Affective touch led to a decrease in false reports of feeling the tactile pulse (FA) during the SSDT. Results in FA were partially mirrored by results in *d`*. There was a tendency towards a main effect of group on *d*’, and exploratory analyses showed that sensitivity in detecting the tactile pulse (*d`*) was significantly higher after receiving affective touch (CT optimal group) in comparison to after receiving non-affective touch (non-CT optimal group). It should be noted, however, that there was no significant group × time interaction for *d’*. Nevertheless, these results showed that participants receiving affective touch were then less inclined to erroneously report the tactile pulse when absent (FA). Alongside, in line with significant results in FA, the CT optimal group also showed a tendency towards a better discrimination of detecting when the tactile pulse was administered from when it was not (*d`*). Therefore, according to our expectations, affective touch was shown to induce a partial increase in tactile accuracy.

To date, this is the first study demonstrating an off-line effect of affective touch on perceptual accuracy and visuo-tactile, multisensory integration. Prior studies have indicated that in a multisensory context (i.e., during multisensory integration tasks) affective touch was perceived as more meaningful when compared to non-affective discriminative touch [18, 72]. During the Rubber Hand Illusion (RHI) or the Enfacement Illusion, for example, affective touch has been linked to a stronger experience of the illusion and an enhancement of body ownership [20–23]. According to these results, it has been argued that CT-afferents may play a unique role in the construction of the bodily self, that is the multimodal perceptually integrated model of one’s own body [18, 20, 25]. However, it remained unclear whether affective touch could induce subsequent alterations in multisensory integration and body perception. This study aimed to fill in this gap in the current literature. Results showed that affective touch can indeed induce a subsequent off-line effect on a multisensory integration task that is the SSDT by increasing participants’ tactile accuracy.

In this respect, it is worth noting that from a neurophysiological point of view, CT-afferents have been linked to the activation of brain areas that are involved in the elaboration and integration of multisensory information (e.g., the posterior insula; [13, 14]), and in the codification of the sense of a bodily self (e.g., the angular gyrus; [3]). Therefore, it could be the case that receiving affective touch alters the way subsequent sensory information are coded and integrated in the building of the bodily self. Specifically, affective touch may increase awareness about one’s own body and therefore determine a subsequent more accurate integration of multisensory information.

This effect was specific for affective touch; non-affective touch did not impact on FA and *d`*. Conversely, non-affective touch induced a partial decrease in the correct detection of touch (HR) during the SSDT. Indeed, the non-CT optimal group showed a significant effect of the Light on HR only after receiving the touch manipulation, with higher HR in trials during which the light was present as compared to trials in which the light was absent. However, this effect was driven by the fact that after the touch manipulation, in the non-CT optimal group there was a decrease in HR in trials during which the light was absent, suggesting that participants receiving non-affective touch were then less able to perceive the tactile pulse without the prompt of the light.

It is possible that the fast touch (30 cm/s) administered in the non-CT optimal group desensitized the same tactile channels required to detect the vibrotactile stimulation administered during the SSDT. Indeed, previous research has shown mechanoceptors to have receptive fields that can vary and adapt according to circumstances (e.g., exposure to visual information and/or sustained stimulation; see [73, 74]). In turn, adaptation of receptive fields has been shown to be related to impaired performances in the detection of subthreshold stimuli due to an increase in the tactile threshold [73–75]. In this respect, a previous psychophysiological study indicated that adaptation to prolonged fast/vibrating tactile stimuli induced a desensitization of skin mechanoreceptors that were then less sensitive in discriminating different textures [75, 76]. Therefore, it could be hypothesised that a similar mechanism influenced participants responses in this study. Specifically, receiving a prolonged sustained tactile stimulation (non-affective fast touch) targeting the same receptive fields involved in the detection of subthreshold touch during the SSDT may have caused an increase in participants’ tactile threshold and therefore a worse detection (i.e., desensitization) of the tactile pulse during the SSDT. According to this explanation, in a previous study by Mirams and colleagues [35] participants showed decreased touch reports during the SSDT after performing a grating orientation task which involved focusing on external touch. Coherently to our reasoning, it could be the case that also in this previous study, the tactile stimulation involved in discriminating grating orientations fatigued and desensitized skin mechanoreceptors, leading to a decrease in touch reports during the subsequent SSDT.

Further SSDT results showed that, in line with previous literature, the presence of the light facilitated participants perception of the tactile pulse during the task. This was reflected by overall higher HR and *d`*, and lower *c* in trials during which the light was present. Contrary to our expectations, the touch manipulation, in both the conditions of affective and non-affective touch, was found not to alter this effect of the light on touch detection during the SSDT. Although the non-CT optimal group showed an effect of the light (higher HR in light-present vs light-absent trials) only after receiving non-affective touch, as previously discussed, this effect seemed to be driven by a decrease in HR in trials during which the light was absent, which can be linked to a desensitization of mechanoreceptors after receiving a fast touch.

In contrast with the original SSDT study [28] the presence of the light was not found to increase false reports of touch. However, this is consistent with some other previous studies which investigated the effect of the light on SSDT performance under different experimental conditions [68, 77], overall suggesting that the presence of the light can influence both correct and incorrect reports of touch. Further research should focus on whether participants’ states or personality traits play a role in determining whether the presence of the light influence more correct or incorrect report of touch during the SSDT.

Throughout the experiment, Skin Conductance Levels (SCLs) were also recorded to analyse whether the touch manipulation induced changes in autonomic physiological arousal. Results indicated that the touch manipulation induced an increase in arousal that was slightly higher for affective vs non-affective touch. After the touch manipulation, during the second repetition of the SSDT, the level of arousal slowly decreased. However, SCLs remained significantly higher compared to baseline levels (during the first repetition of the SSDT). A single study only previously analysed the relationship between affective touch and SCLs with results similar to the results of this study [36]. Indeed, Ree and colleagues [36] showed SCLs to increase after receiving a tactile stimulation, with a greater increase for CT optimal vs non-CT optimal touch, although this difference did not reach significance. However, it should be noted that other studies using different indexes of arousal, such as the heart rate, the heart rate variability, and the amplitude in skin conductance response, all suggested affective touch to be associated with a decrease, rather than an increase in arousal [53, 55, 78]. Therefore, a comprehensive picture of how different sympathetic and parasympathetic indexes of arousal vary due to affective touch is still to be drawn by future research.

Interestingly, SCLs were positively correlated with participants’ ability to correctly detect the tactile pulse during the SSDT (HR), and to accurately discriminate when the tactile pulse was administered and when it was not (*d`*). These results are in agreement with results of a previous study from our research group, in which the change scores in SCLs between experimental conditions was found to be associated with a parallel increment in participants ability to discriminate touch (*d`*) in light-present trials of the SSDT [29]. Taken together, these findings suggest that participants’ responses during the SSDT can be influenced by their arousal levels, with higher SCLs predicting a more accurate tactile perception. Moreover, SCLs were found to increase more steeply during affective touch as compared to non-affective touch. Therefore, it could be hypothesized that SCLs may mediate the relationship between the touch manipulation and tactile accuracy, with affective touch being associated with higher SCLs and increased tactile accuracy. Future research should further investigate this hypothesis.

In line with our expectations, affective touch was found to induce an increase in positive mood (Happiness and Calmness) and a decrease in negative mood (Anxiety and Sadness). However, and in contrast to our expectation, this effect was not specific for affective touch, but was also present after receiving non-affective touch. Mood was partially influenced by participants’ perceived quality of touch during the touch manipulation (Intensity and Pleasantness). Indeed, participants reported feeling calmer and less anxious the more pleasant they had rated the touch manipulation, and reported feeling sadder the more intense the touch manipulation was perceived to feel. However, mood and perceived quality of touch were found not to influence SCLs.

To summarize, results of this study showed for the first time an off-line effect of affective touch on visuo-tactile, multisensory integration and perceptual awareness. In the context of the SSDT, where participants are required to detect a tactile pulse independently from the presence/absence of a concomitant light flashing, this effect translated in a greater tactile accuracy. Moreover, our results suggested SCLs to be a possible mediating mechanism linking affective touch to a greater tactile accuracy during the SSDT. As previously explained, from a neurophysiological point of view, CT-afferents have been linked to the activation of brain areas that are involved in body awareness and in the building of the sense of a bodily self through the integration of multisensory information (e.g., the posterior insula; [13, 14] and the angular gyrus; [3]). Therefore, it could be the case that an activation of this brain network after receiving affective touch increases body awareness altering the way subsequent sensory information are coded and integrated in the building of the bodily self. Specifically, results of this study might suggest that by enhancing the sense of a bodily self through affective touch, body perception may benefit from a greater accuracy and awareness. In other words, affective touch may increase awareness about one’s own body as indicated by a more accurate elaboration of multisensory information during the SSDT.

However, this study is not without limitations. For example, it could be argued that repeating the SSDT could affect participants’ performance during the task. However, previous research has shown participants’ responses during the SSDT to be consistent over time (i.e., multiple repetitions) with no learning processes involved [31]. Moreover, it should be noted that the two experimental groups of this study presented with a significant difference in the way they were influenced by the light in the correct detection of touch during the SSDT at baseline (before the experimental manipulation). The reason for this difference in baseline is not clear. Although no other between-groups differences were found at baseline. To overcome this issue, future research may benefit from the use of a within-subject design that minimizes possible biases linked to between-subjects differences. An alternative implementation of the current design would be to repeat this study adding an additional experimental group undergoing a no touch or a very slow touch (e.g., 0.3 cm/s) manipulation. Although in this case the risk of between-subjects differences at baseline would increase, an additional experimental condition would allow to control for specificity of results. For example, it would allow us to determine whether our interpretation of results in the non-CT optimal group as being linked to desensitization of mechanoceptors is correct. Following this stream of research, another adjustment that could be used to control for pre-existing between-groups differences would be to adopt a linear mixed model analysis with participants’ responses at baseline used as covariates.

Nonetheless, conclusions of the current research promote a possible use of affective touch as an intervention for those pathological conditions in which perception of the body is altered. Previous studies have shown affective touch to increase the sense of body ownership in neuropsychological conditions such as asomatognosia and somatoparaphrenia [72, 79, 80]. A future stream of research should investigate whether the use of affective touch as an intervention can be expanded also to those psychopathological disorders characterized by alterations in multisensory integration and perceptual accuracy such eating disorders, body dysmorphic disorders, and medically unexplained symptoms.

## Supporting information

S1 Dataset Affective touch and perceptual accuracy. (XLSX)
